# Detoxifying effect of *Nelumbo nucifera* and *Aegle marmelos* on hematological parameters of Common Carp (*Cyprinus carpio* L.)

**DOI:** 10.2478/v10102-010-0052-9

**Published:** 2010-12

**Authors:** Rajamanickam Vinodhini

**Affiliations:** Aquatic Biodiversity Research Center, Department of Advanced Zoology and Biotechnology, St. Xavier's College (Autonomous), Palayamkottai, Tamilnadu, India

**Keywords:** water quality, hemoglobin, glucose, Nelumbo nucifera, Aegle marmelos, common carp

## Abstract

The objective of this study was to investigate the efficacy of *Nelumbo nucifera* and *Aegle marmelos* on common carp exposed to sub-lethal concentrations of combined heavy metals (5 ppm) under laboratory conditions. The fish were treated with *Nelumbo nucifera* (500 mg/kg bwt) and *Aegle marmelos* (500 mg/kgbwt) for 30 days as a dietary supplement. The blood biochemical parameters of the fish were evaluated by analyzing the level of red blood cells (RBC), packed cell volume (PCV), hemoglobin concentration, glucose, cholesterol, iron and copper. The findings of the present investigation showed significant increase in hemoglobin (*p<*0.001), RBC (*p<*0.01) and PCV (*p<*0.01) of herbal drug-treated groups compared with metal-exposed fish. Conversely, glucose and cholesterol level in blood of common carp showed significant reduction compared with heavy-metal-exposed groups. All the values measured in *Nelumbo nucifera* and *Aegle marmelos* treated fish were restored comparably to control fish. Our results confirmed that *Nelumbo nucifera* and *Aegle marmelos* provide a detoxification mechanism for heavy metals in common carp.

## Introduction

Worldwide interest in food hygiene has increased in recent years. In order to improve public health, authorities of most countries now pay great attention to the production of ecologically pure and nutritious food for human consumption. Efforts have been taken in all stages of processed food to avoid contamination with various pollutants (Staniskiene & Garaleviciene, 2004). As defined by WHO, health is a state of complete physical, mental and social well-being and not merely the absence of disease or infirmity. The world's growing population and the need to maximize agricultural production has given rise to the need to produce human food from aquatic resources that include fish production (FAO, 1998; Kureshy *et al*., 2000). Fish is an essential and irreplaceable food in diet. Fish contain protein, polyunsaturated fatty acids, micronutrients, vitamin A and minerals. A major part of the world's food is being supplied from fish sources, making it essential to secure the health of fishes (Tripathi *et al*., 2002).

Fish are not only a major ecosystem component, making it important to study their physiological response mechanism when confronted with environmental stress (Wu *et al*., 2002). Any environmental disturbance can be considered as a potential source of stress as it promotes a number of responses in the fish to deal with the physiological changes triggered by exterior challenges (Martinez *et al*., 2002). These responses can be detected in fish in the form of changes in biomarkers, alteration in erythrocytes such as cell volume and enzyme activities (Vinodhini & Narayanan, 2008b; Vinodhini & Narayanan, 2009c; Donaldson, 1981; Jee & Kang, 2005). Of all aquatic fauna, fish is the most susceptible to the effects of heavy metal toxicants (Nwaedozie, 1998; Agbozu *et al*., 2007) among other aquatic organisms. Because of their toxicity, long survival time, circular course in the biosphere and accumulation in nature, heavy metals are of prime importance among stress generators to fish (Vinodhini & Narayanan, 2009a). Heavy metals cause differences in the physiological and chemical properties of fish blood (Hughes *et al*., 1988). Our previous study reported the bioaccumulation of combined heavy metals in common carp under sublethal levels of metal exposure (Vinodhini & Narayanan, 2008a). Internal perturbation in fish as a result of exposure to heavy metals alters their aerobic metabolism and their survival can be threatened, with death as the final result in some cases.

In view of the above discussion, the bioaccumulation of heavy metals in fish cannot be entirely avoided but there is a clear need for such pollutants to be minimized with the aim of reducing both direct and indirect impacts on human health (SCAN, 2003). It is a widely acceptable statement that endogenous production of defensive proteins is not always effective with increasing exposure to heavy metals, so an exogenous supply of nutrients will find important roles in diminishing the cumulative effects of toxic metals. Nowadays, new biochemical research is focusing on the use of whole-plant-derived dietary supplements, phytochemicals and provitamins that assist in maintaining good health and combating disease, reffered to as functional foods or nutraceuticals (Hoareau and Dasilva, 1999). In several industrialized societies, plant-derived prescription drugs constitute an element in the maintenance of health (UNESCO, 1996). The prophylactic and therapeutic effects of plant food extracts are increasingly being recognized as potential health promoters in both man and livestock (Bizimenyera *et al*., 2007). In this regard, plants need scientific validation and exploration before used as phytotherapeutic agents in control of acute and chronic disorders. Two such medicinal plants, *Nelumbo nucifera* and *Aegle marmelos,* were previously recognized for their remarkable therapeutic effect in the management of various diseases and in the ayurvedha and siddha system of medicine they were selected as herbal drugs. The aim of the present study was to evaluate the efficacy and detoxification effect of *Nelumbo nucifera* and *Aegle marmelos* in an aquatic system exposed to 5 ppm combined heavy metals, using common carp as an experimental model.

## Methods

Fresh water common carp weighing about 35.75 ± 0.60 g were acquired from a local fish aquarium from the southern district of Tamilnadu, India. Fish were individually examined for external necrosis, infection and parasites. Those which proved to be free from pathological signs were retained for the study. The feeding frequency, length and body weight of the fish and physiochemical characteristics of water used for acclimation in control and heavy metals exposed groups during day and night time were recorded (Vinodhini & Narayanan, 2009b). The institutional ethical committee guides for the care and use of laboratory animals were followed.

### Selection of plant material

Fresh flower petals of *Nelumbo nucifera* (Nymphaceae) and leaves of *Aegle marmelos* (Rutaceae) were collected from south India, Tamilnadu. The leaves and flower petals were washed with tap water to remove sand, dirt and then rinsed thoroughly with distilled water. The plant parts were dried with constant weight in an oven at 45°C for 48 hours. They were pulverized to fine powder using electrical grinder and shaken through test sieves of 200 mm diameter and aperture 250 µm to collect the fine powder and then labeled in plastic polyethylene bottles. This crude powder of *Nelumbo nucifera* and *Aegle marmelos* serves as herbal drug. Silymarin (refined powder) was purchased in a local drug house and used as standard in this study.

### Experimental design

The fish were divided into eight groups (Table 1). Group1 was the control group and received only fish feed throughout the experiment. Group (G2–G5) fish were exposed to sublethal concentration of 5 ppm of combined heavy metals such as cadmium, chromium, nickel and lead for 32 days. The chronic exposure period and blood biochemical changes in carp were reported (Vinodhini & Narayanan, 2009b). At the end of the 32^nd^ day of metal exposure period, the fish from holding tanks in group 5 were further subdivided into groups (G6–G8) (n=10). Group 6 fish were treated with crude *Nelumbo nucifera* (500 mg/kg/bwt), group 7 were treated with crude *Aegle marmelos* (500 mg/kg/bwt) and group 8 with standard Silymarin (100 mg/kg/bwt). They were mixed in commercial fish feed (1 kg) each separately by a mechanical grinding system and molded into small pellets. Fish in groups G6–G8 were fed with these herbal drugs at a rate of 3–4% of body weight twice a day for 30 days by floating feed technique.

**Table 1:** Classification of heavy-metal-exposed and drug-treated fish groups.

**Groups**	**Heavy metal exposure days**	**Groups**	**Drug treatment**
G1	Control		
G2	1	G6	*Nelumbo nucifera*
G3	8	G7	*Aegle marmelos*
G4	16	G8	Silymarin
G5	32		

No mortality was observed among the control and herbal drug treated groups. The body weight of the fish was measured at the start and the end of the drug treatment period. At the end of the drug treatment period fish from control and drug-treated groups (G6–G8) were assessed for measurement of blood parameters. The fish were caught gently in a small net avoiding stress as much as possible. They were sacrificed and blood samples were collected to analyze the hematology and biochemical profile. The hematological parameters including hemoglobin (Drabkin & Austin, 1935), blood glucose by glucose oxidase method (Bergmeyer & Bernt, 1974), total cholesterol by the method of Allain (1974) and Young (1997), red blood cells by Ochei & Kolhatkar (2005) and packed cell volume by Wintrobe's tube method (Ochei & Kolhatkar, 2005). The concentrations of iron (detection limit 0.005 ng/mL) and copper (detection limit 0.002 ng/mL) in serum samples were determined by using graphite atomic absorption spectroscopy method (Monisov, 1992). All statistical results were expressed in mean ± standard deviation. Values of *p<*0.05, *p<*0.01 and *p<*0.001 were considered as slightly significant, significant and highly significant, respectively.

## Results

**Figure 1:**
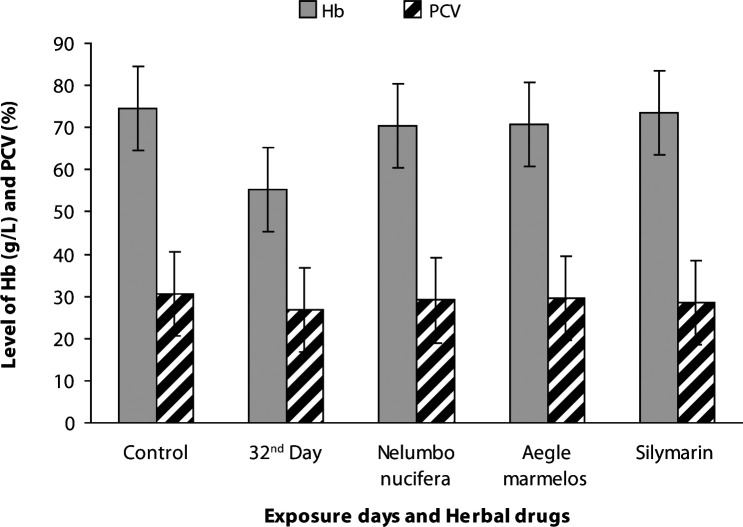
Levels of haemoglobin (***p<0.001) and packed cell volume (**p<0.01) in control, heavy metal exposed and drug treated common carp.

**Figure 2:**
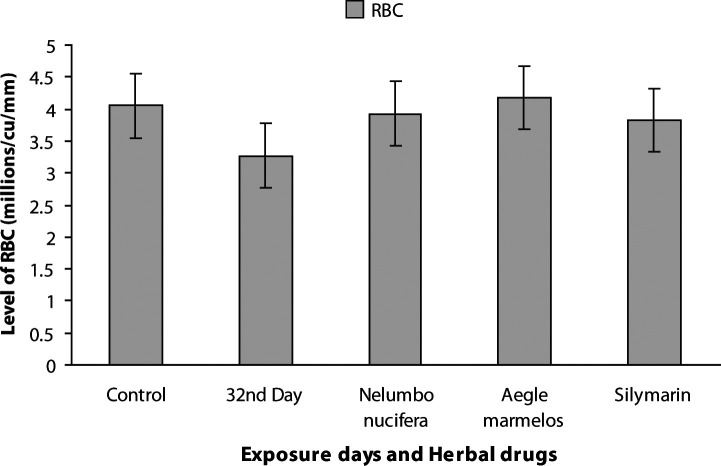
Levels of red blood cells (**p<0.01) in control, heavy metal exposed and drug treated common carp.

**Figure 3:**
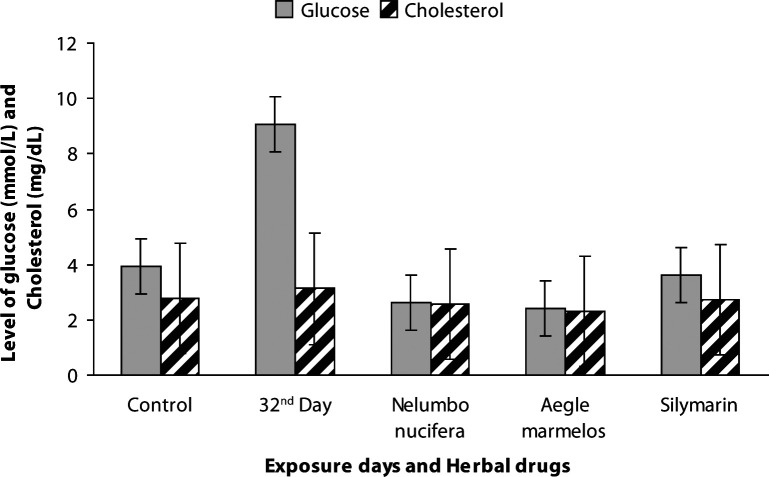
Levels of blood glucose (***p<0.001) and cholesterol (**p<0.01) in control, heavy metal exposed and drug treated common carp.

**Figure 4:**
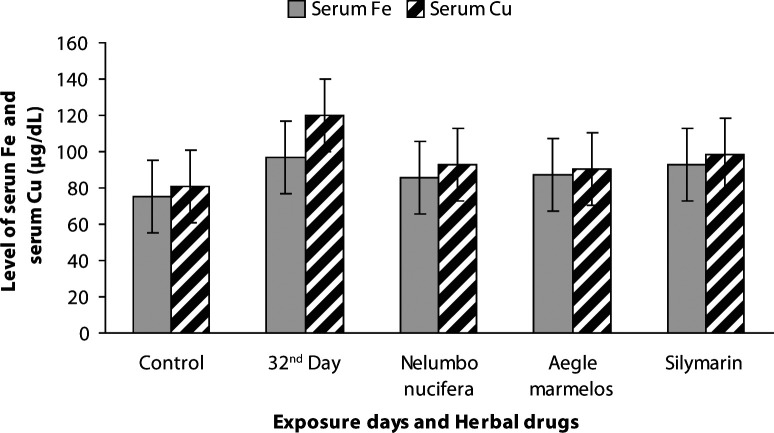
Levels of serum iron (***p<0.001) and copper (***p<0.001) in control, heavy metal exposed and drug treated common carp.

The physicochemical parameters of the water in the herbal drug-treated groups (G6–G8) are presented in Table 2. The average values of total length and body weight of carp were 11.54 ± 0.61 cm and 30.66 ± 2.90 g for control, 14.52 ± 0.19 cm and 42.50 ± 0.23 g for *Nelumbo nucifera*, 12.68 ± 0.18 cm and 38.46 ± 0.30 g for *Aegle marmelos* and 14.34 ± 0.17 cm and 40.64 ± 0.21 g for Silymarin. The hematological results in freshwater carps treated with herbal drugs (G6–G8) showed a significant increase in packed cell volume (*p<*0.01), hemoglobin concentration (*p<*0.001) and RBC (*p<*0.01) and are presented in detail in figures 1 and 2. Blood biochemical markers such as glucose, cholesterol, iron and copper showed significant reduction (*p<*0.001, *p<*0.01, *p<*0.001, *p<*0.001) in herbal-drug-treated carps (G6–G8) compared with metals intoxicated groups. The values measured in drug-treated fish (G6–G8) were near to control fish values (Figures 3–4).

**Table 2:** Water quality parameters measured in the experimental ponds during the drug treatment period (*p<*0.05).

Water Quality Parameter	Tank I	Tank II	Tank III
Temperature (°C)	27 ± 0.1	27.2 ± 0.1	26.5 ± 0.1
pH	7.6 ± 0.15	7.45 ± 0.2	7.62 ± 0.3
Electrical conductivity (μS/cm)	1455 ± 5	1425 ± 5	1485 ± 5
Total dissolved solids (mg/L)	945 ± 5	965 ± 5	972 ± 5
Alkalinity (mg/L)	175 ± 2.5	182 ± 3.5	190 ± 3.0
Total hardness (mg/L)	320 ± 3.2	325 ± 2.5	340 ± 2.5
Dissolved oxygen (mg/L)	6.5 ± 0.5	6.7 ± 0.5	6.2 ± 0.56
Total ammonia (mg/L)	17.5 ± 1.5	18.2 ± 1.7	18.5 ± 1.5
Salinity (%)	14.37 ± 0.17	14.25 ± 0.5	14.80 ± 1.5

Tank I: *Nelumbo nucifera* treatment Tank II: *Aegle marmelos* treatment Tank III: Silymarin treatment

## Discussion

Hematological studies in fish can provide information on the effect of the external environment on the internal physiology of fish. Biochemical changes in blood values are particularly important to diagnose disease and stress in fish (Vinodhini & Narayanan, 2008c; Ranzani Paiva *et al*., 2003; Rehulka *et al*., 2004). A detailed evaluation of hematological parameters serves as a sensitive index for controlling fish diseases and improving fish cultivation (Canzanave *et al*., 2005). Most of the research papers dealt with the information regarding the toxic effect of single or multiple heavy metals and their hazardous effect on the health of aquatic organisms (Ovie & Ubogu, 2008; Agbozu *et al*., 2007; Jee & Kang, 2005). Our previous study demonstrated the obvious toxic effect of heavy metals and their hematological alterations in Cyprinus carpio (Vinodhini & Narayanan, 2009b). The present study focused on the detoxicating effect of *Nelumbo nucifera* (500 mg/kg/bwt), *Aegle marmelos* (500 mg/kg/bwt) and Silymarin (100 mg/kg/bwt) in common carp exposed to 5 ppm of combined heavy metals.

A high statistical significance (*p<*0.001) in the increased concentration of hemoglobin was found in all drug-treated fish (G6–G8). Our previous study reported a significant reduction in blood hemoglobin levels of heavy metals exposed fish. The results of our present experiment suggest that the herbal drugs influence the hemotopoietic system for hemoglobin production. Physiologically, hemoglobin acts as a blood buffering agent and oxygen carrier in blood. Packed cell volume (PCV) is a major hematological characteristic that changes with fish activity (Anyanwu *et al*., 2007). A statistically significant (*p<*0.01) increase in packed cell volume of fish treated with herbal drugs (G6–G8) was observed compared with heavy metal exposed fish. The behavioral responses such as slow swimming and limited appetite observed during metal exposure period were reverted in herbal drug-treated fish (G6–G8). The rapid swimming response and intake of herbal drugs as feed mix confirmed the aerobic metabolism due to normal cellular function.

A slightly significant increase (*p<*0.01) in RBC of drug-treated groups (G6–G8) observed in the present report strengthens our views that both *Nelumbo nucifera* and *Aegle marmelos* triggered the increase in red blood cells. The increase in RBC indicates a compensatory response to meet oxygen demands. Our previous study reported a decrease in red blood cells of heavy metal exposed fish which developed anemia. The values of hemoglobin, packed cell volume and red blood cells of *Nelumbo nucifera* and *Aegle marmelos* treated fish were in the normal ranges of control and standard drug-treated groups. Our results are in agreement with the increased hematological parameters in hybrid catfish fed with boiled jackbean seed meal (Osuigwe *et al*., 2005). Blood glucose was used as an indicator of environmental stress (Shilbergeld, 1974). The glucose level in our drug-treated groups (G6–G8) showed highly significant reduction (*p<*0.001) compared with metal-exposed fish. Our previous reports evidenced an increase in blood glucose with characteristic hyperglycemia in heavy-metal-exposed fish. The decrease of blood glucose level in herbal drug-treated groups (G6–G8) might be due to the mode of action of herbals that convert excess glucose into reserve fuel glycogen by stimulating glycogenesis mechanisms for future demands.

Blood cholesterol was slightly but significantly (*p<*0.01) reduced in herbal-drug-treated fish (G6–G8). The increased cholesterol level observed in the heavy-metal-exposed common carp showed a decreased and maintained cholesterol level in drug-treated fish (G6–G8) comparable to the control fish pointing out the metabolic adjustments in fish influenced by herbal drugs. The concentration of iron and copper in serum of drug-treated fish (G6–G8) showed a significant reduction (*p<*0.001) after a 30-day treatment period. Our previous study showed a significant increase in the iron and copper levels of heavy-metal- exposed carps. The levels of iron and copper in herbal-drug-treated fish were found to be nearly the same as those of normal fish; this finding shows the positive effect of herbal drugs in mitigating the toxicity induced by heavy metals. Herbal drugs act as effective modulators in reducing the toxicity and increasing the immunological tolerance of fish (Vinodhini & Narayanan, 2009d). Based on the above results, the present study confirmed that *Nelumbo nucifera* and *Aegle marmelos* exert a significant hematological effect compared to standard Silymarin and control fish. The above observed effect in drug-treated fishmight be due to the higher level of nutritional factors in aquatic weeds (Kalita *et al*., 2007).

## Conclusion

We suggest that the two herbal drugs *Nelumbo nucifera* and *Aegle marmelos* were effective in combating the toxic effect of heavy metals and more efficient in relieving the stress induced by heavy metals. Where heavy metals are present, the cost of applying these herbals could be reduced by their incorporation into the feed mixture to promote fish health. In this way aquatic species could be protected from the effect of heavy metals. Further exploration is needed to determine the response to *Nelumbo nucifera* and *Aegle marmelos* in other fish varieties.
